# Updates to the Autism Intervention Research Network on Physical Health (AIR-P) Research Agenda

**DOI:** 10.7759/cureus.44388

**Published:** 2023-08-30

**Authors:** Emily Hotez, Madeline Haley, Julian A Martinez-Agosto, Jeffrey Anderson, Heather Brown, Kristen Choi, Lisa A Croen, Patrick Dwyer, Priyanka Fernandes, Dena Gassner, Morénike Giwa Onaiwu, Candace M Gragnani, Laura Graham Holmes, Steven Kapp, Dana Kim, Maria Massolo, Brianna Montgomery, Heini M Natri, Julianna A Rava, Kashia A Rosenau, Jeffrey Roth, Dawn Rudolph, Jackie G Ryan, Paul Shattuck, Lindsay Shea, Zachary J Williams, Rujuta B Wilson, Alice Kuo

**Affiliations:** 1 Department of Medicine, University of California Los Angeles, Los Angeles, USA; 2 Department of Educational Psychology, Faculty of Education, Edmonton, CAN; 3 Department of Nursing, School of Nursing, University of California Los Angeles, Los Angeles, USA; 4 Department of Health Policy and Management, Fielding School of Public Health, University of California Los Angeles, Los Angeles, USA; 5 Division of Research, Kaiser Permanente Northern California, Oakland, USA; 6 Laboratory of Neurocognitive Development, Center for Mind and Brain, University of California Davis, Davis, USA; 7 Department of Health Sciences, School of Social Work, Adelphi University, New York, USA; 8 School of Humanities, University of East Anglia, Norwich, GBR; 9 Department of Pediatrics, University of California Los Angeles, Los Angeles, USA; 10 Department of Social Work, School of Public Health, Boston University, Boston, USA; 11 Department of Psychology, University of Portsmouth, Portsmouth, GBR; 12 Department of Research, Association of University Centers on Disabilities, Silver Spring, USA; 13 Department of Research, Kaiser Permanente Northern California, Oakland, USA; 14 Department of Computational Science, Translational Genomics Research Institute, Part of City of Hope Cancer Center, Phoenix, USA; 15 Department of Technical Assistance & Network Engagement, Association of University Centers on Disabilities, Silver Springs, USA; 16 Department of Neurodiversity Strategy and Education, University of Alberta, Faculty of Rehabilitation Medicine, Edmonton, USA; 17 Department of Policy Research, Mathematica, Princeton, USA; 18 Center of Policy and Analytics, A.J. Drexel Autism Institute, Drexel University, Philadelphia, USA; 19 Department of Hearing & Speech Sciences, Vanderbilt Brain Institute, Vanderbilt University, Nashville, USA; 20 Department of Psychiatry, Semel Institute for Neuroscience and Human Behavior, Los Angeles, USA; 21 Department of Preventive Medicine, David Geffen School of Medicine, University of California Los Angeles, Los Angeles, USA

**Keywords:** mental health, health, neurodiversity, research, autism

## Abstract

Introduction: Autistic individuals, now representing one in 36 individuals in the U.S., experience disproportionate physical health challenges relative to non-autistic individuals. The Health Resources and Services Administration's (HRSA) Autism Intervention Research Network on Physical Health (AIR-P) is an interdisciplinary, multi-center Research Network that aims to increase the health, well-being, and quality of life of autistic individuals. The current paper builds on the initial AIR-P Research Agenda (proposed in Year 1) and provides an updated vision for the Network.

Methods: Updates to the Research Agenda were made via the administration of a Qualtrics survey, and disseminated widely to all AIR-P entities, including the Research Node Leaders, Steering Committee, Autistic Researcher Review Board, and collaborating academic and non-academic entities. Network members were tasked with evaluating the Year 1 Research Agenda and proposing additional priorities.

Results: Within each Research Node, all Year 1 priorities were endorsed as continued priorities for research on autism and physical health. Specific topics, including co-occurring conditions and self-determination, advocacy, and decision-making, were particularly endorsed. Opportunities for exploratory studies and intervention research were identified across Research Nodes. Qualitative responses providing feedback on additional research priorities were collected.

Conclusion: The updated AIR-P Research Agenda represents an important step toward enacting large-scale health promotion efforts for autistic individuals across the lifespan. This updated agenda builds on efforts to catalyze autism research in historically underrepresented topic areas while adopting a neurodiversity-oriented approach to health promotion.

## Introduction

Autistic individuals, currently one in 36 (2.8%) [1], demonstrate disproportionate rates of co-occurring and chronic conditions, including cardiovascular diseases, diabetes, epilepsy, psychiatric conditions, gastrointestinal disorders, and mobility challenges. They also experience disproportionate psychosocial challenges, low self-reported quality of life, and a low life expectancy [2-9]. Health challenges in autistic populations are due to myriad factors, including genetic predispositions and co-occurring conditions, barriers to high-quality healthcare services and supports, including high administrative burden and resulting service fragmentation, and persistent exposure to implicit and explicit stigma and stress across interpersonal, educational, healthcare, and other contexts [10,11].

In light of these disparities, the Autism Intervention Research Network on Physical Health (AIR-P) seeks to establish and maintain a research network to enhance the physical health and well-being of autistic children, adolescents, and adults, particularly underserved and vulnerable individuals and families. The AIR-P is funded by the Health Resources and Services Administration Maternal and Child Health Bureau (MCHB) and places a strong emphasis on developing network priorities in partnership with autistic community members, service providers, family members, advocates, and academics.

Background and context

The initial (Year 1) Research Agenda process involved collaborations among select entities within the AIR-P through a three-pronged methodology. This included: 1) Ideation and design: Through a series of planning meetings, the National Coordinating Center (NCC) and Steering Committee (SC) identified preliminary priority domains based on their areas of expertise and background knowledge of the literature; 2) Literature review and synthesis: The NCC and SC created a targeted annotated bibliography of quantitative and qualitative research in the identified domains and synthesized the findings in a literature review to refine the domains; and 3) Network engagement: Experts across the network engaged in a two-step process via Qualtrics surveys with 24 AIR-P members to identify priority research topics within each domain based on three criteria (need and urgency; research impact; and person- and family-centeredness).

The initial Research Agenda process resulted in the Year 1 Research Agenda which comprised six core research priority domains: 1) Primary Care Services and Quality; 2) Community-Based Lifestyle Interventions; 3) Gender, Sexuality, and Reproductive Health; 4) Health Systems and Services; 5) Neurology; and 6) Genetics. The Research Agenda also comprised four cross-cutting priorities that applied across all domains: 1) neurodiversity-oriented care; 2) facilitating developmental transitions; 3) methodologically rigorous interventions; and 4) health disparities.

The current study

The current paper describes updates to the Year 1 AIR-P Research Agenda. Specifically, this research aimed to 1) assess continued endorsement of Research Agenda priorities; and 2) identify emerging priorities for subsequent years of the cooperative agreement. Our efforts to update the Year 1 Research Agenda included an added focus on the specific research activities necessary to advance topical research priorities, including exploratory studies, intervention research, and big data approaches. These activities were added to ensure that the Network and all members of the autism community committed to advancing research that improves the health, well-being, and quality of life of autistic individuals can become better equipped to translate research priorities into new studies. In addition to this change, the AIR-P has since expanded and further developed collaborations across the Network; as such, the updated Research Agenda sought to reflect the perspectives of a wider range of the AIR-P community. The AIR-P Research Agenda will be iteratively revised as the field continues to advance to ensure that ongoing efforts within the Network reflect state-of-the-art research, practice, and policy priorities.

## Materials and methods

The current study used a cross-sectional, web-based Qualtrics survey administered to the AIR-P with the purpose of updating the initial Research Agenda, proposed in Year 1 and published in Kuo et al. (2022) [10]. All AIR-P members were invited to participate in the survey.

Sample recruitment and eligibility

There were two waves of recruitment. We first focused on obtaining responses from our proximal Network. The AIR-P entities are listed in Table 1.

**Table 1:** AIR-P entities *These entities were involved in the Year 1 Research Agenda Process. All of the entities listed above were involved in efforts in the current study to update the Research Agenda. AIR-P: Autism Intervention Research Network on Physical Health

Entity*	Description
National Coordinating Center (NCC)*	The NCC oversees the collaboration efforts of all entities specified below. The NCC has been instrumental in the design and implementation of innovative and impactful autism research by building and supporting an infrastructure that enables collaborative research activities among diverse groups of experts.
Node Leaders*	Autism research leaders across the country with expertise in each Node topic:1) Primary Care Services and Quality; 2) Community-Based Lifestyle Interventions; 3) Gender, Sexuality, and Reproductive Health; 4) Health Systems and Services; 5) Neurology; and 6) Genetics
Autistic Researcher Review Board (ARRB)*	A committee of autistic scholars provides Network guidance and consultation for research studies within the Network.
Steering Committee Members*	A committee is comprised of the NCC, Node leaders, ARRB representatives, and technical assistance entities. The focus of this committee is to guide Network decisions to promote growth and innovation.
Scholars	The Scholars include early-career researchers seeking to promote the health and well-being of autistic individuals across the lifespan through research. They receive pilot and feasibility funding for their proposed projects, mentorship and training, and professional development and collaboration opportunities.
Collaborating research entities (CRE)	Fifteen U.S. research institutions participate in Network activities, including leading research studies, supporting Scholars, and contributing general guidance, support, and consultation throughout the Network.
Affiliated Government/Non-profit Organizations	Membership organizations that support and promote a national network of university-based interdisciplinary programs, including University Centers of Excellence in Developmental Disabilities (UCEDDs), Leadership Education in Neurodevelopmental Disabilities (LENDs), Intellectual and Developmental Disabilities Research Centers (IDDRCs), and Developmental-Behavioral Pediatrics (DBP) programs.

Our response rate over a two-week period for the National Coordinating Center (NCC), SC, Autistic Researcher Review Board (ARRB), Scholars, and collaborating research entities (CREs) was 31/72 (43%). To solicit additional feedback, we invited those who were distally affiliated with the AIR-P as webinar attendees or LISTSERV members. The survey was disseminated via email and was open for an additional two weeks to all eligible participants (n = 884). With this broader recruitment strategy, we had 89 survey respondents (10% response rate).

Measures

The Qualtrics survey was developed through both informal and formal collaborative synchronous and asynchronous discussions occurring via the Zoom platform and e-mail among the AIR-P entities (Table 1).

A collaborative approach to survey development was useful in determining the optimal methodology for ascertaining continuous or emerging priorities within the Network. Generally speaking, the AIR-P elected to garner feedback on the Year 1 AIR-P Research Agenda as written, but select updates were made accordingly. As an example, the Year 1 Research Agenda identified three Primary Care priorities; in the updated Research Agenda survey, one of these priorities was disaggregated into two priorities in order to glean more specific and informative feedback. The Genetics priority areas were revised upon collaborations with the ARRB. The survey included the following specific assessments:

Continued Node Priority Area Endorsements

Priority areas identified from the Year 1 Research Agenda were re-assessed for each Node using five-point Likert scale items. Participants were asked to indicate the extent to which they agreed that each priority area remains an important priority for the AIR-P. Three to five topics per research Node were included. Network endorsement of each topic was determined based on whether more than 50% of respondents responded "agree" or "strongly agree" for a particular item.

Priority Research Activities

Participants were asked to select the top two research activity priorities for each Node. Options were derived from Node Leader consensus and included three types of research activities: 1) intervention research; 2) exploratory studies; 3) linked datasets and big data analytic approaches.

Additional Research Priorities

Within each Research Node topic, participants were invited to provide open-ended responses about additional research priorities that should be considered for the AIR-P Research Agenda.

## Results

Sample

The sample characteristics are reported in Table 2.

**Table 2:** Demographic and sample characteristics *The demographics items were at the end of the survey; Of the 89 who responded, 70 completed the survey (79% completion rate). MPH: Master of Public Health; MA: Master of Arts; MS: Master of Science; Ph.D.: Doctor of Philosophy; MD: Doctor of Medicine; PsyD: Doctor of Psychology; AIR-P: Autism Intervention Research Network on Physical Health; ARRB: Autistic Researcher Review Board; NCC: National Coordinating Center; CRE: collaborating research entities

Characteristic	n (N = 70)*	%
Gender		
Male	16	22.90%
Female	52	74.30%
Prefer not to answer	2	2.90%
Race		
White	48	68.60%
Black or African American	7	10.00%
Asian	4	5.70%
More than one race	1	1.40%
Not listed above	1	1.40%
Prefer not to answer	9	12.90%
Ethnicity		
Hispanic	6	8.60%
Non-Hispanic	58	82.90%
Prefer not to answer	6	8.60%
Highest level of education		
High school level	3	4.30%
Bachelor's level	5	7.10%
Master's level (MPH, MA, MS, etc.)	10	14.30%
Doctorate level (Ph.D., MD, PsyD, DO, etc.)	45	64.30%
Other	4	5.70%
Prefer not to answer	3	4.30%
Years of experience in the field since receiving the highest level of education		
0-2 years	8	11.40%
3-5 years	16	22.90%
6-10 years	13	18.60%
10-20 years	21	30.00%
Over 20 years	11	15.70%
Autistic		
Yes	16	22.90%
No	51	72.90%
Prefer not to answer	3	4.30%
Do you have an autistic family member or a close friend?		
Yes	36	51.40%
No	31	44.30%
Prefer not to answer	3	4.30%
Are you a caregiver of an autistic individual?		
Yes	15	21.40%
No	22	31.40%
Prefer not to answer / Missing	33	47.10%
AIR-P role (select all that apply)		
Steering Committee/Node Leader	4	5.70%
ARRB member	9	12.90%
NCC	2	2.90%
Scholar	6	8.60%
Government/Nonprofit member	6	8.60%
CRE member	11	15.70%
Community member	20	28.60%
Healthcare practitioner	19	27.10%
Researcher	44	62.90%
Student	4	5.70%

The full sample included 89 interdisciplinary professionals and individuals with lived experience affiliated with the AIR-P. Within the full sample, 70 completed the survey in its entirety, including the demographic items at the end of the survey (79% completion rate). To confirm that the survey responses did not significantly differ between those who completed all survey questions versus those who did not, we checked the first set of priority items within each sub-group and confirmed that the findings were consistent across both groups.

The sample was comprised of CRE members (15.7%); ARRB members (12.9%); AIR-P Scholars (8.6%); government and non-profit organization members (8.6%); SC members (5.7%), who were also Research Node Leaders (5.7%); and NCC members (2.9%). The sample identified as researchers (62.9%), community members (28.6%), healthcare practitioners (27.1%), and students (5.7%).

The participants were primarily female (females: 74.3%; males: 22.9%; 2.9% preferred not to answer:) and non-Hispanic (non-Hispanic: 82.9%; Hispanic: 8.6%). The sample consisted of the following race breakdown: White (68.6%), Black/African American (10.0%), Asian (5.7%), more than one race (1.7%), not listed (1.4%); 12.9% preferred not to answer (12.9%). Participants’ highest level of education included: doctorate level (64.3%), master’s level (14.3%), bachelor’s level (7.1%), high school (4.3%), other (5.7%); 4.3% preferred not to answer (4.3%). Participants indicated their number of years of experience in the field since their highest degree: 0-two years (11.4%), three-five years (22.9%), six-10 years (18.6%), 10-20 years (30.0%), and over 20 years (15.7%).

The sample included respondents who identified as autistic (22.9%), a family member or close friend of an autistic individual (51.4%), or a caregiver of an autistic individual (21.4%). Importantly, several participants identified with more than one of these categories, with 16% identifying as both autistic and a family member or close friend of an autistic individual and 4% identifying as an autistic caregiver.

Continued node priority area endorsements

Continued AIR-P priorities with examples are presented in Table 3.

**Table 3:** The Network's continued priorities (topical) *Findings revealed continued endorsement of all, based on >50% responding “agree” or “strongly agree”. ADHD: attention deficit hyperactivity disorder; LGBTQ+: lesbian, gay, bisexual, transgender, queer or questioning, intersex, asexual, and more; PCP: primary care physician

Node/Topic remains an important priority for the AIR-P Research Network*	Strongly disagree/disagree		Neither agree nor disagree		Strongly agree/agree	
	n	%	n	%	n	%
Primary Care Services and Quality (N = 89)						
Primary care health service delivery, e.g., primary care provider training models, primary care office modifications	4	4.5%	8	9.0%	74	83.1%
Clinical care quality, e.g., specialized protocols for autistic individuals, additional clinical services	3	3.4%	3	3.4%	83	93.3%
Self-determination/advocacy/decision-making, e.g., autistic individuals' active engagement in healthcare	3	3.4%	3	3.4%	79	88.8%
Co-occurring mental health conditions, e.g., ADHD, anxiety, depression, stress-induced	1	1.1%	4	4.5%	83	93.3%
Community-Based Lifestyle Interventions (n = 82)						
Nutrition/ physical activity, e.g., engaging families and individuals in activity; increasing community support; strengthening the evidence base	3	3.7%	13	15.9%	63	76.8%
Relationships/social connectedness/community participation, e.g., interventions/supports that engage communities, families, children, and peers; friendships and relationships	3	3.7%	5	6.1%	71	86.6%
Quality of life/ health promotion, e.g., holistic approaches to wellness/thriving	3	3.7%	8	9.8%	68	82.9%
Self-determination/ advocacy/ decision-making, e.g., skill development and autonomy; understanding needs and potential environmental adaptations	1	1.1%	8	9.8%	70	85.4%
Adaptive skill development, e.g., hands-on training in life skills	3	3.7%	13	15.9%	63	76.8%
Gender, Sexuality, and Reproductive Health (n=73)						
Sexual education/knowledge, e.g., developing sexual health curricula for autistic individuals, promoting awareness and knowledge	2	2.7%	7	9.6%	62	84.9%
Intersectionality/LGBTQ+/gender identity; e.g., strategies for supporting youth; understanding prevalence, needs, and experiences of LGBTQ+ autistic individuals	2	2.7%	14	19.2%	55	75.3%
Vulnerable/marginalized/diverse populations, e.g., sex and gender disparities	3	4.1%	9	12.3%	59	80.8%
Family/parent education and support, e.g., individual, parent, and family education on sexual health; understanding caregiver needs	2	2.7%	3	4.1%	66	90.4%
Physician training and quality care provision, e.g., physician training on sexual health/care for autistic individuals	2	2.7%	7	9.6%	62	84.9%
Self-determination/advocacy/decision-making, e.g., self-determination around sexual behavior	1	1.4%	6	8.2%	64	87.7%
Health Systems and Services (n = 70)						
Transitions (general, e.g., developing transition resources; facilitating major health care transitions	2	2.9%	9	12.9%	55	78.6%
Navigating systems and service models, e.g., health care navigation for adolescents and adults; models of effective transition practices	1	1.4%	5	7.1%	61	87.1%
Physician training and quality care provision, e.g., preparing PCPs in the adult health care system to serve the autistic population	1	1.4%	4	5.7%	61	87.1%
Family support, e.g., educating families and individuals about transitions and adult care	3	4.3%	4	5.7%	60	85.7%
Co-occurring mental health conditions, e.g., facilitating psychiatric health care transitions	1	1.4%	4	5.7%	63	90.0%
Neurology (n = 70)						
Co-occurring conditions, e.g., epilepsy, motor difficulties, sensory issues, sleep difficulties	1	1.4%		8.6%	58	82.9%
Adult neurologic outcomes, e.g., transition of care, co-occurring conditions, trajectories/development	4	5.7%		10.0%	53	75.7%
Care coordination, e.g., decision trees for primary care and neurologists for referrals and tests; integrating neurologists with other health care providers	1	1.4%		10.0%	56	80.0%
Genetics (n = 70)						
Exploring the role of rare genetic variants in co-occurring conditions, e.g., Understanding genetics and obesity, co-occurring motor conditions, adaptive functioning, etc., medical treatments individualized to specific genes	14	20.0%	8	11.4%	44	62.9%
Research on improving genetic counseling outcomes, e.g., training genetic counselors to work with autistic individuals and their families	6	8.6%	13	18.6%	47	67.1%
Ethical issues surrounding autism genetics research, e.g., addressing the autistic community’s concerns about genetics research; moving away from cure/cause research towards increasing quality of life	5	7.1%	3	4.3%	55	78.6%

**Figure 1:**
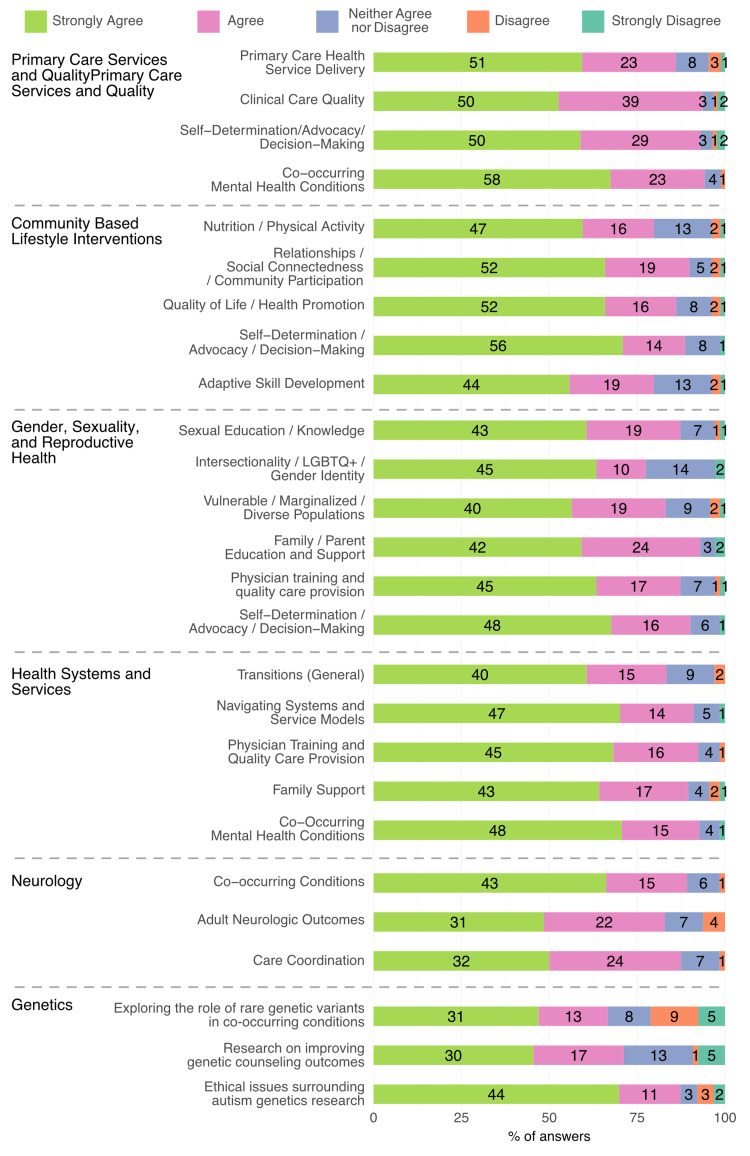
The Network's continued priorities (topical) LGBTQ+: lesbian, gay, bisexual, transgender, queer or questioning, intersex, asexual, and more

The findings are presented by Research Node. This information is graphically depicted in Figure 1.

All of the priorities asked about on the survey, derived from the Year 1 survey, were endorsed as continued priorities (as demonstrated by more than 50% indicating "agree" or "strongly agree" that the topic remains an important priority for the network). Within each Node, the following priorities received the highest proportion of "strongly agree" or "agree" responses: Primary Care Services and Quality: co-occurring mental health conditions (93.3%) and clinical care quality (93.3%); Community-Based Lifestyle Interventions: relationships and social connectedness (86.6%) and self-determination, advocacy, and decision-making (85.4%); Gender, Sexuality, and Reproductive Health: family education (90.4%) and self-determination, advocacy, and decision-making (87.7%); Health Systems and Services: co-occurring mental health conditions (90.0%), navigating systems and service models (87.1%) and physician training and quality care provision (87.1%); Neurology: co-occurring conditions (82.9%) and care coordination (80.0%); and Genetics: ethical issues surrounding autism genetics research (78.6%) and research on improving genetic counseling outcomes (67.1%).

Priority research activities

The survey included an assessment of participants’ top two highest-ranked methodological priorities for each of the topical priorities specified above (Table 4).

**Table 4:** The highest-ranked Network priorities (methodological) *Options to select research activities were not mutually exclusive. LGBTQ+: lesbian, gay, bisexual, transgender, queer or questioning, intersex, asexual, and more

	Interventions		Exploratory studies		Linked datasets and big data	
Node/Priority*	n	%	n	%	n	%
Primary Care Services and Quality (n = 89)						
Primary care health service delivery	57	66.3%	41	47.7%	25	29.1%
Clinical care quality	56	65.1%	43	50.0%	29	33.7%
Self-determination/advocacy/decision-making	46	53.5%	62	72.1%	17	19.8%
Co-occurring mental health conditions	57	66.3%	45	52.3%	33	38.4%
Community-Based Lifestyle Interventions (n = 82)						
Nutrition/physical activity	54	65.9%	42	51.2%	21	25.6%
Relationships/social connectedness/community participation	53	64.6%	52	63.4%	14	17.1%
Quality of life/health promotion	42	51.2%	43	52.4%	29	35.4%
Self-determination/advocacy/decision-making	51	62.2%	54	65.9%	18	22.0%
Adaptive skill development	63	76.8%	38	46.3%	16	19.5%
Gender, Sexuality, and Reproductive Health (n = 73)						
Sexual education/knowledge	47	64.4%	41	56.2%	14	19.2%
Intersectionality/LGBTQ+ /gender identity	29	39.7%	47	64.4%	28	38.4%
Vulnerable/marginalized/diverse populations	22	30.1%	43	58.9%	34	46.6%
Family/parent education and support	46	63.0%	40	54.8%	18	24.7%
Physician training and quality care provision	49	67.1%	38	52.1%	23	31.5%
Self-determination/advocacy/decision-making	43	58.9%	44	60.3%	17	23.3%
Health Systems and Services (n = 70)						
Transitions (general)	--	--	47	67.1%	43	61.4%
Navigating systems and service models	--	--	48	68.6%	35	50.0%
Physician training and quality care provision	--	--	48	68.6%	31	44.3%
Family support	--	--	51	72.9%	29	41.4%
Co-occurring mental health conditions	--	--	52	74.3%	43	61.4%
Neurology (n = 70)						
Co-occurring conditions	38	54.3%	31	44.3%	30	42.9%
Adult neurologic outcomes	32	45.7%	34	48.6%	29	41.4%
Care coordination	32	45.7%	34	48.6%	29	41.4%
Genetics (n = 70)						
Explore the role of rare genetic variants in co-occurring conditions, their natural history, and their responsiveness to therapeutic interventions	12	17.1%	26	37.1%	43	61.4%
Improving genetic counseling outcomes by enhancing genetic counselor and physician-patient communication	28	40.0%	37	52.9%	11	15.7%
Ethical Issues surrounding autism genetics research (data ownership, consenting, prenatal screening)	17	24.3%	47	67.1%	15	21.4%

Options included (a) intervention research, (b) exploratory studies, and (c) linked datasets and big data approaches. For Primary Care Services and Quality intervention research was the highest priority for research on primary care health service delivery (66.3%), co-occurring mental health conditions (66.3%), and clinical care quality (65.1%). Exploratory studies were the highest priority for research on self-determination, advocacy, and decision-making (72.1%).

For Community-Based Lifestyle Interventions, interventions research of the highest priority was for adaptive skill development (76.8%); nutrition and physical activity (65.9%); and relationships, social connectedness, and community participation (64.6%); exploratory studies were also highly endorsed for this priority (52; 63.4%). Exploratory studies were the highest priority for research on self-determination, advocacy, and decision-making (65.9%); interventions were also highly endorsed for this priority (51; 62.2%) and quality of life and health promotion (52.4%).

For Gender, Sexuality, and Reproductive Health, interventions were the highest priority for physician training and quality care provision (67.1%); sexual education and knowledge (64.4%); and family and parent education and support (63.0%). Exploratory studies were the highest priorities for research on intersectionality (64.4%); self-determination, advocacy, and decision-making (60.3%); interventions were also highly endorsed for this priority (43; 58.9%); and vulnerable, marginalized, and diverse populations (58.9%).

Prior to survey dissemination, there was consensus among the Health Systems and Services Research Node to focus efforts more on exploratory studies and big data approaches than intervention research, given the Node capacities and resources. Between those two methodological options, respondents indicated exploratory studies as the highest priorities for research on co-occurring mental health conditions (74.3%); family support (72.9%); navigating systems and service models (68.6%), physician training and quality care provision (68.6%); and general healthcare transitions (67.1%).

For Neurology, interventions were selected as the highest priorities for research on co-occurring conditions (54.3%). Exploratory studies were identified as the highest priorities for adult neurologic outcomes (48.6%) and care coordination (48.6%).

For Genetics, linked and big data approaches were identified as the highest priorities for research on exploring the role of rare genetic variants in co-occurring conditions (61.4%). Exploratory studies were the highest priority for research on ethical issues surrounding autism genetics research (67.1%) and improving genetic counseling outcomes (52.9%).

Additional research priorities

We obtained qualitative responses from participants on additional research priorities that should be considered within the Network (Table 5).

**Table 5:** Open-ended responses to additional priorities ESL: English as a second language; PTSD: post-traumatic stress disorder

Node / Priority	Example Topics
Primary Care Services and Quality	● Co-occurring physical conditions (e.g., gastrointestinal, asthma, allergy, concerns)
	● Intersectionality (e.g., autistic racial and gender minority and low-income patients), particularly issues related to mis-/late diagnosis; diagnosis and stigma
	● Identifying ways to enhance primary care’s role in autism, rather than relying on specialists
	● Improving clinical care, particularly related to communication, accessibility, and assistive technology as well supported-decision-making in primary care
	● Inclusion and prioritization of autistic adults
	● Trauma-informed care
	● Enhancing systems and services, including improving access to community-based services; development of primary care models to facilitate transition and bridge sub-specialty care; and telehealth
Community-Based Lifestyle Interventions	● Making interventions more accessible, including using universal design
	● Specialized types of interventions, including aquatic, art, and music therapy as well as social-media interventions
	● The intersection of interventions and summer camps
	● Improving the sense of community for autistic individuals, through community training and awareness campaigns, particularly to reduce and prevent camouflaging and burnout
	● Interventions specifically for autistic adults, including work and employment training, daily living support beyond life skills
	● Service provider training
	● Making interventions more appropriate for culturally diverse families, particularly for ESL and low-income families
	● Integrating more holistic approaches to health in interventions
Gender, Sexuality, and Reproductive Health	● Making gender, sexuality, and reproductive health education more accessible
	● Training teachers to provide more effective education
	● Family planning and pregnancy support, particularly for those with co-occurring intellectual disabilities
	● The intersection of health and policy for autistic individuals, particularly restrictions on abortion access
	● Developmental issues, including puberty and menopause
	● Issues of consent in relationships
	● Support for learning about one’s sexual orientation and gender identity and finding community
	● The role of trauma in gender, sexuality, and reproductive health
Health Systems and Services	● Accessibility in health systems and services, including addressing systemic barriers to accessibility
	● Diagnostic overshadowing and disability discrimination
	● The role of early and childhood interventions in providing resources and supports for adults
	● Staff and service provider education to increase confidence in supporting autistic individuals, particularly autistic individuals with chronic conditions
	● Guidelines for effective transition, including trauma-informed transition services
	● Interagency collaboration to connect young adults with other systems (e.g., Medicaid) and support different outcomes (e.g., employment) as well as the use of patient navigators
	● Pathways for diagnosis and treatment of co-occurring psychiatric conditions
Neurology	● Strategies for making neurology services more accessible
	● Aging-related and/or other chronic neurological conditions in autistic individuals
	● Brain-gut connection
	● Enhancing the capacity of neurologists to support autistic patients
	● Enhancing understanding of families’ perspectives and priorities related to neurological care
Genetics	● Enhancing the accessibility and awareness of genetics services
	● The genetic linkages among autism, PTSD, and trauma
	● Availability and accessibility of genetics counseling
	● Ensuring genetics research provides valuable information and support to individuals and families
	● Increasing input from families on genetics services
	● Physical health issues in autistic people with rare genetic disorders
	● Training practitioners at all levels regarding the role of genetics for autistic patients
Updated Cross-Node Priorities	● Making interventions more accessible
	● Prioritizing intersectionality
	● Developmental / lifespan considerations
	● Provider/physician training
	● Person-family-centeredness in research
	● Trauma-informed care

Select priorities emerged multiple times, and we considered these priorities to be updated cross-Node priorities: making interventions more accessible, prioritizing intersectionality, developmental and lifespan considerations, provider and physician training, person-and family-centeredness in research, and trauma-informed care.

Updated AIR-P research agenda

All findings led to the updated Research Agenda (Table 6).

**Table 6:** The updated Research Agenda of the Autism Intervention Research Network on Physical Health (AIR-P) ^1^ Based on Network revisions prior to survey administration (generated through informal consensus) and survey feedback. ^2^ Example topics within each priority area are provided in Table 3.

Updated research priorities^1^	Methodological priorities		
Node/Priority^2^	Interventions	Exploratory studies	Linked data and big data
Primary Care Services and Quality			
Primary care health service delivery	X		
Clinical care quality	X		
Self-determination/advocacy/decision-making		X	
Co-occurring mental health conditions	X		
Community-Based Lifestyle Interventions			
Nutrition/physical activity	X		
Relationships/social connectedness/community participation	X		
Quality of life/health promotion		X	
Self-determination/advocacy /decision-making		X	
Adaptive skill development	X		
Health Systems and Services			
Transitions (general)		X	
Navigating systems and service models		X	
Physician training and quality care provision		X	
Family support		X	
Co-occurring mental health conditions		X	
Gender, sexuality, and reproductive health			
Sexual education/knowledge	X		
Intersectionality/LGBTQ+/gender identity		X	
Vulnerable/marginalized/diverse populations		X	
Family/parent education and support	X		
Physician training and quality care provision	X		
Self-determination/advocacy/decision-making		X	
Neurology			
Co-occurring conditions	X		
Adult neurologic outcomes		X	
Care coordination		X	
Genetics			
Exploring the role of rare genetic variants in co-occurring conditions			X
Research on improving genetic counseling outcomes		X	
Ethical issues surrounding autism genetics research		X	
Updated Cross-Node Priorities	Making interventions more accessible prioritizing intersectionality developmental/lifespan considerations provider/physician training person-family-centeredness in research trauma-informed care		

## Discussion

The Health Resources and Services Administration (HRSA) AIR-P is an interdisciplinary, multicenter research network that aims to increase the health, well-being, and quality of life of autistic individuals. The current paper builds on the initial development of the AIR-P Research Agenda (proposed in Year 1) [10] and provides an updated vision for the Network. This study yielded several findings.

Continued node priority area endorsements

There was a continued endorsement of all Research Node priorities identified in the development of the Year 1 AIR-P Research Agenda. This finding suggests that each of the six AIR-P Research Nodes representing six physical health topics relevant to autistic populations is supported by diverse members of the autism research community.

In addition to confirming continued endorsement of each Research Node, we were able to identify priority topics, determined by the highest proportion of participant endorsement. Within Primary Care, Health Systems and Services, and Neurology, co-occurring conditions were among the highest priorities. It is well established that co-occurring conditions, including anxiety, depression, sleep-wake disorders, and others, are more prevalent in the autistic population than in the general population [12]. The state of the evidence on the mechanisms underlying these differences is inconclusive, yet consistently underscores a confluence of interacting individual and environmental factors. The majority endorsed the need for interventions targeting co-occurring mental health conditions in autistic individuals. Indeed, although the focus of the AIR-P is predominantly centered on physical health, there is wide consensus in the evidence base that mental and physical health are inextricably linked and there is a need for holistic assessment, evaluation, and care.

Within both Community-Based Lifestyle Interventions and Gender, Sexuality, and Reproductive Health, self-determination, advocacy, and decision-making emerged as some of the most highly endorsed priorities. This finding aligns with research that highlights the importance of these capacities for autistic individuals across the life course [13, 14]. Despite the burgeoning evidence base on the utility of self-advocacy, there remains a paucity of research on self-advocacy as it pertains to physical health (e.g., agency and self-efficacy in making doctor’s appointments, setting health goals, navigating insurance, and implementing health-promoting lifestyle strategies), which is particularly critical in light of the systemic barriers that challenge these capacities for this population (e.g., healthcare fragmentation and lack of provider training).

Additional exploratory research on health-related self-advocacy can complement existing initiatives geared towards promoting health-related self-advocacy, including the Academic-Autistic Spectrum Partnership in Research and Education (AASPIRE) toolkits [15], efforts within the Autistic Self-Advocacy Network, and the Special Olympics [16], as well as inform the development of new interventions. It will be critical to spur additional partnerships led by groups with multiple marginalized intersectional identities to ensure that health-related self-advocacy initiatives directly address the unique circumstances of diverse families.

Within Genetics, ethical issues surrounding autism genetics research emerged as the highest priority, with a strong need identified for exploratory research. Indeed, exploitation and abuse often occur in genetics research on marginalized populations, including those with developmental differences [17]. There is increasing attention to the myriad ethical issues in genomic medicine, particularly in the context of neurodevelopmental traits [17]. Imperatively, genetic autism research must result in research benefits for autistic people, accomplished by minimizing risk and potential harm and enhancing well-being and self-determination [17]. While parents’ and clinicians’ views on genetic counseling have been surveyed [17], information on autistic people’s needs, concerns, and views regarding genetic testing and genetic counseling is lacking. Existing studies on genetic counseling for autism focus on transgenerational risk and the genetic diagnosis of autism. However, as endorsed by survey participants, research is needed on the genetic underpinnings of conditions that disproportionately affect autistic people, as well as on genetic counseling and the communication of genetic information. Generally speaking, these results are consistent with our Year 1 Research Agenda, which elucidated support within the Network for identifying genes linked to co-occurring conditions or treatment responses, addressing the autistic community’s concerns about genetics research, and moving away from cure and cause research toward increasing quality of life.

Priority research activities

In addition to the research activities required to further study co-occurring mental health challenges and self-advocacy, the Network emphasized the need for exploratory studies on a range of topics. These included intentionally prioritizing vulnerable, marginalized, and diverse populations. For example, neurological outcomes among autistic adults, often de-prioritized relative to younger age groups, were specifically endorsed. Exploratory studies were also endorsed for studying intersectionality, a theoretical framework for understanding how social categorizations (gender, class, disability, race, and others) operate to disadvantage people and legitimize power [18]. Public health often conceptualizes marginalized groups as monolithic, rather than recognizing the myriad ways in which multiple disadvantaged identities interact and compound to negatively affect lifelong health [18]. Social injustices magnified by the COVID-19 pandemic spurred a renewed recognition of the importance of intersectionality in understanding and addressing health disparities, and it is possible that our findings reflect this trend [19].

Exploratory research was also a high priority for healthcare and coordination topics. As an example, exploratory studies were cited as important for research on all topics within the Health Systems and Services Node, including general healthcare transitions, navigating systems and service models, physician training and quality care provision, family support, and co-occurring mental health conditions, as well as care coordination within the Neurology Node. This is consistent with research that finds high healthcare fragmentation, low healthcare quality, administrative burden, and a resounding need in the field for service coordination models and healthcare provider training that meet the needs of autistic individuals and their families [20].

Participants identified potential opportunities for intervention studies across all Nodes (with the exception of the Health Systems and Services and Genetics Nodes). The Primary Care, Community-Based Lifestyle, and Gender Nodes revealed the greatest number of potential intervention opportunities. Across each of these Nodes, interventions included individual-level (e.g., identifying educational, psychosocial, or other strategies to promote active engagement in healthcare among autistic individuals and their families) and systemic (e.g., testing innovative training or care models) interventions.

In the context of the Genetics Node, almost two-thirds endorsed the need for methodological approaches that utilized linked datasets and big data. Large-scale genetic testing and tracking within databases have the potential to inform research and clinical practice. Such datasets can help identify specific gene contributions and interactions while serving to advance the diagnostic yield of genetic testing [21]. When implemented to promote self-determination and well-being, these approaches have the potential to positively contribute to the quality of life of autistic individuals. The AIR-P Genetics Node is uniquely poised to help facilitate the creation of registries for health conditions that disproportionately affect autistic people and will continue to advance these efforts.

Importantly, big data approaches were not cited as a priority research activity beyond the Genetics Node. This finding contrasts with other studies that indicate a need for large-scale, population-level data analysis to account for the lack of socioeconomically diverse samples in autism research. Our results may, in large part, be attributable to the lack of large, high-quality datasets in autism and physical health research. Indeed, research suggests the need for greater data infrastructure (e.g., improved linkages among national, regional, and local data sources) to increase researchers’ capacity to leverage existing data, improve power, and conduct hypothesis testing. To be sure, big data approaches have the potential to capture the health experiences of diverse and underserved autistic individuals and produce findings that are translatable to public health and health equity initiatives.

Additional research priorities

We obtained qualitative responses from participants on additional research priorities that should be considered within the Network. The data revealed potential areas of focus for each Node. There were also priorities that emerged multiple times that we considered to be updated cross-Node priorities: making interventions more accessible, prioritizing intersectionality, developmental and lifespan considerations, provider and physician training, person- and family-centeredness in research, and trauma-informed care. Although we didn’t specifically assess participants’ endorsement of the Year 1 cross-cutting themes, including neurodiversity-oriented care, facilitating developmental transitions, methodologically rigorous intervention studies, and addressing health disparities, the qualitative findings from the current study clearly align with and expand on these previously identified themes. Moving forward, it is anticipated that research that prioritizes cross-Node themes will lead to research that is maximally beneficial for autistic populations.

Updated AIR-P research agenda and its implications

All findings coalesced into the updated Research Agenda. The purpose of delineating the AIR-P Research Agenda in the current study was twofold: 1) to spur research in alignment with the priorities of both interdisciplinary experts in the field and individuals with lived experience; and 2) more broadly, to serve as the impetus for more generalized research, practice, and policy transformation. Such transformations include promoting the inclusion of autistic individuals as both researchers and research participants (research) [22]; preventing and addressing implicit and explicit stigma that both indirectly and directly bars autistic individuals from quality care (practice) [23]; and dismantling administrative burden that perpetuates fragmented healthcare (policy) [11]. The AIR-P will continue to be responsive to emerging priorities in the field moving forward and consider the Research Agenda as a continuously evolving blueprint for the next steps.

Limitations

This research had several limitations. Although we captured a range of perspectives, our survey was in-depth, time-consuming, and administered without participant incentive, which impeded our ability to collect data from a large sample. Further, our full response rate, albeit low, is also imprecise, given the challenges of parsing out the full reach of our recruitment efforts through listservs and the anonymity of our survey. As reliance on wide-scale recruitment tools such as social media continues to increase in research, it will be exceedingly important for researchers to employ more precise tracking.

Due to our restricted sample, there were instances in which the highest endorsed research priority in a particular category was only endorsed by one or two participants more than the second and/or third-highest endorsed research priorities. Future research should address priorities that were highly endorsed but perhaps not discussed in detail in our Discussion section, as well as priorities that emerged through our open-ended items.

Another limitation is that the rationale underlying a particular endorsement was not assessed in this research. This shortcoming could have critical implications for interpreting these findings, as endorsement could potentially represent 1) that a particular research topic is important or 2) that current research on a particular topic is low-quality or exploitative to the autistic community, and significant efforts are required to improve research in this area. This may be particularly salient for controversial areas of research, such as genetics [21]. The general nature of the survey also impeded our capacity to report on research activity priorities with a higher level of granularity; for example, exploratory studies may include participatory action research, qualitative methodologies, and ethnographic approaches, which have been identified in previous autism research as critical, or could simply refer to quantitative studies that seek to summarize particular topics. Further research should seek to discern greater detail in this regard.

An advantage of our research was that we were able to survey experts in the field of autism research. Given this facet of our research, the majority of our sample had obtained doctoral degrees. Additional research, however, is necessary to capture large samples more reflective of diverse autism professional communities. Further, although our survey sampled interdisciplinary autism professionals and we had a 100% response rate from the ARRB, the individuals with lived experience in our sample were often also researchers or professionals within the autism community. Future research should prioritize perspectives from individuals, caregivers, and family members who do not have autism-related professions to ensure that research is driven by these communities. This will have myriad downstream effects, including providing instrumental and social resources to those who have been particularly marginalized within the healthcare system by administrative burden, stigma, and other factors [11].

## Conclusions

The updated AIR-P Research Agenda represents an important step forward for continuing to enact large-scale health promotion efforts for autistic individuals across the lifespan. This updated Agenda will build on efforts to catalyze autism research in historically underrepresented topic areas while adopting a neurodiversity-oriented approach.
